# Do we age faster in absence of gravity?

**DOI:** 10.3389/fphys.2013.00134

**Published:** 2013-06-05

**Authors:** Camillo Di Giulio

**Affiliations:** Department of Neurosciences and Imaging, University of ChietiChieti, Italy

Our anatomy and body functions are related to gravity, we are the result of our gravity. Neurons, bones, muscles, and the whole support system were all developed in response to the gravity. The way we grow, we run, we adapt and the development of diseases depends on gravity force (Aubert et al., 2005). Enzymes and gene expression are the result of the interaction of the mechanical forces with our structures (Heather et al., 2006). The correlations between life-span and gravity are important to understand our physiological limit. In space, muscle atrophy is a physiological adaptation due to a loss of muscle proteins that would contribute to an acceleration in aging. The correlations between life-span and gravity seem to generate ideas to understand our physiological limits. Living in space for months or years would promote physiological adaptations that represent the bases in gravitational physiology. In space muscle atrophy rate is approximately 5% per month, and VO_2_ max is reduced by 25% after few a weeks of stay (Narici and De Boer, 2011). The reduction in leg volume is due to muscle atrophy and fluid loss. Muscle biopsies in astronauts have shown that after 5 days in microgravity, the mean cross sectional area of muscle fiber was reduced. The reduction in muscle mass is accompanied by reduced force (Raja et al., 2010). Our genes needs a level of physical activity (Booth et al., 2002), from birth the body require gravity to grow (Silver et al., 2003). Considering that the relation between cytoplasm and mitochondrial mass defines the tissue oxygen consumption, several similarities seem to exist among absence of gravity, altitude-hypoxia, and aging, which are all conditions characterized by a common reduction in maximum oxygen consumption, in lactic capacity and in mitochondrial volume. Therefore, chronic cellular hypoxia itself could be one of the causes responsible for the reduction of muscle mass and sarcopenia in absence of gravity and in the elderly. Skeletal muscle inactivity is associated with a loss in muscle proteins and reduced force-generating capacity (Biolo et al., 2003; Vernikos and Schneider, 2010).

In altitude, in microgravity and in prolonged bed rest there is a loss of muscle proteins that seems to promote an acceleration in aging. The loss of proteins in space and in aging seems to mimic the adverse changes comparing with prolonged bed rest (Mohler, 1962; Powers et al., 2007). With loss of muscle, CO_2_ production is lower, so in microgravity the ventilatory responses to hypoxia and hypercapnia are modified (Prisk et al., 2000). Is oxidative stress the key regulator of cells signaling pathways and damage to ROS accumulation that is responsible for promoting aging?

Muscle Hypoxic Inducible Factor (HIF) and gravity have to be correlated in particular with the decrease in muscle pO_2_ during exercise. Indeed, muscle intracellular pO_2_ falls to very low levels during exercise (3–4 Torr), which leads to muscle HIF accumulation. HIF is an essential factor in maintaining ATP levels in cells inducing activation of VEGF, and is implicated in muscle fatigue (Seagroves et al., 2001). During endurance training, the exercising skeletal muscle experiences severe and repetitive oxygen stress and inadequate O_2_ supply or hypoxia, which seem to promote angiogenesis similar to that occurring in microgravity. The importance of exercising in space in preventing weight loss due to dehydration and orthostatic intolerance result in the development of countermeasures aimed at preventing sarcopenia. Astronauts undergo a strict countermeasure program consisting mostly of physical activity. This process is necessary in longer duration missions like the mission on Mars, which requires about 6 months each way. Considering that muscle pO_2_ supply in astronauts decreases and that ROS increase during space flight, oxidative stress seems to be the key regulator of cell signaling pathways through ROS increase and aging? Moreover HIF as a mediator of muscle adaptation during exercise, a correlation between HIF and ROS has to be hypothesized for astronaut adaptation. Moreover astronauts presenting sarcopenia due to muscle hypoxia, show reduced CO_2_ production, and CO_2_ modulates muscle pH homeostasis that is fundamental for exercising in space. The Italian physiologist Angelo Mosso proposed in 1904 to supply balloonists (aeronauts) with compressed oxygen containing 8% CO_2_ “If at night we feel oppressed all we have to do is move; muscle contraction produces carbonic acid and resets the balance between the gases in the blood” (Di Giulio et al., 2006).

We need a sufficient muscular mass for thermoregulation, so forced training and nutritional supplement are important. The development of countermeasures are necessary for missions of longer duration, like for Mars exploration. The cardiovascular system is also influenced by Microgravity and seems to be an adaptation rather than pathological changes (Antonutto and Di Prampero, 2003; Verheyden et al., 2010). The deterioration of muscle quantity and quality, the negative calcium balance, the osteoporosis (Zhang et al., 2008; Narici and De Boer, 2011), the alteration in the immunological system (Sonnenfeld and Shearer, 2002) and sleep pattern relived in microgravity are similar to the aging effects (Vernikos and Schneider, 2010). If we consider that we were born to move and for physical activity, the increase in VO_2_ max during exercise is age-dependent and with age we became less gravity-dependent and consequently molecules, cells and tissue loose their integrity leading to die for accidents or inactivity.

Microgravity studies cover several areas and the preparation of man exploration of Mars requires a multidisciplinary approach. These studies correlating absence of gravity with age will be helpful for gerontologists that differentiate life-span from healthy-life, which is even more important. The question is if an increase in man life-span would be possible in a space station or whether spending more than 6 months in a space station would affect people's life expectancy. The studies correlating absence of gravity with age and ROS accumulation will be helpful for gerontologists and physiologist, expanding our comprehension of the laws of nature and enrich our life's quality.
